# Supplementary material for: Transient domain boundary drives ultrafast magnetisation reversal
Source: Nat Commun. 2025 Sep 5;16:8233. doi: 10.1038/s41467-025-63571-3 (PMC12413464; doi:10.1038/s41467-025-63571-3)
Supplement: Supplementary file 1 — Supplementary Information [file 41467_2025_63571_MOESM1_ESM.pdf]

SUPPLEMENTARY INFORMATION

Transient domain boundary drives ultrafast magnetisation reversal

Martin Hennecke[1*], Daniel Schick[1*],
Themistoklis P. H. Sidiropoulos[1], Jun-Xiao Lin[2], Zongxia Guo[2],
Grégory Malinowski[2], Maximilian Mattern[1], Lutz Ehrentraut[1],
Martin Schmidbauer[3], Matthias Schnuerer[1],
Clemens von Korff Schmising[1], Stéphane Mangin[2], Michel Hehn[2],
Stefan Eisebitt[1,4]

[1]Max-Born-Institut für Nichtlineare Optik und Kurzzeitspektroskopie, Max-Born-Straße 2A, 12489 Berlin, Germany.
[2]Université de Lorraine, CNRS, Institut Jean Lamour, 2 allée André Guinier – BP 50840, F-54011 Nancy, France.
[3]Leibniz-Institut für Kristallzüchtung, Max-Born-Straße 2, 12489 Berlin, Germany.
[4]Technische Universität Berlin, Institut für Optik und Atomare Physik, Straße des 17. Juni 135, 10623 Berlin, Germany.

*Corresponding author(s). E-mail(s): hennecke@mbi-berlin.de; schick@mbi-berlin.de;

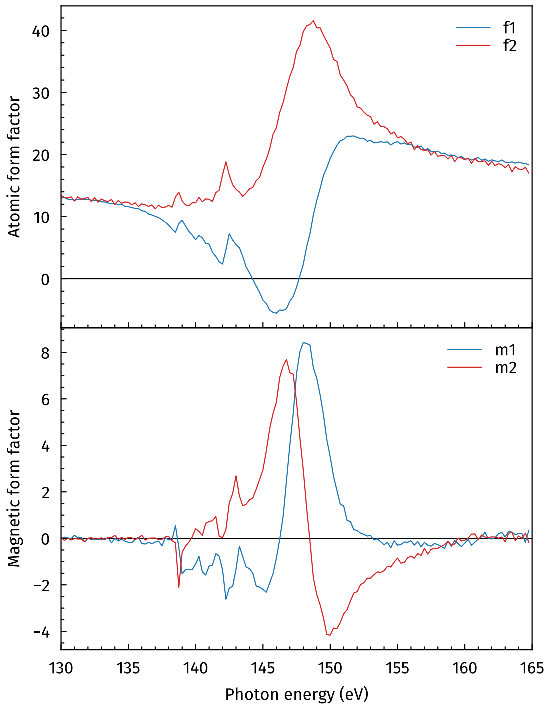

Supplementary Fig. 1 Atomic and magnetic form factors. Atomic (f_1, f_2) and magnetic (m_1, m_2) form factors of the $Gd_{25}Co_{75}$ layer at the Gd $N_{5,4}$ resonance. The spectral dependence of f_2, which is proportional to the imaginary part of the complex refractive index and thus also to the absorption coefficient, shows that the $Gd_{25}Co_{75}$ absorption is highest in the photon energy range of 148–150 eV.

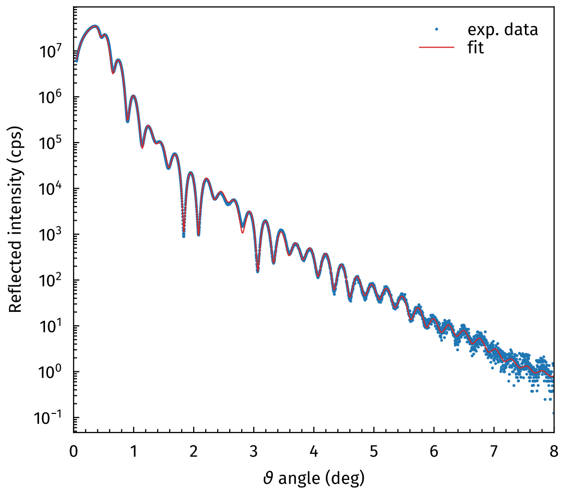

Layer	$t \pm 0.05$ (nm)	$\rho \pm 500$ (kg/m^3)	$\sigma \pm 0.05$ (nm)	ρ_{nom} (kg/m^3)
Pt(1)	0.50	21,400	0.24	21,400
Pt(2)	2.29	19,902	0.08	21,400
Cu(1)	0.55	11,558	0.53	8,960
Cu(2)	1.06	12,723	0.21	8,960
GdCo	9.38	7,900	0.44	7,900
Ta(1)	2.71	14,652	0.33	16,650
Ta(2)	0.89	11,322	0.44	16,650
Si	substrate	2,330	0.26	2,330

Supplementary Fig. 2 Hard-X-ray reflectometry and structural layer parameters. Hard-X-ray specular reflectometry (XSR) measurements (blue dots) on the studied $Gd_{25}Co_{75}$ sample along with a corresponding simulation (red solid line), fitted using the software RCRefSimW [1] to retrieve the structural, non-magnetic properties (thickness t, mass density ρ, rms roughness σ) of the individual layers of the heterostructure. The values of the nominal densities ρ_{nom} are obtained from Ref. [2]. The significantly increased density of the Cu layer can be attributed to the diffusion of heavy atoms from the neighbouring Pt layer.

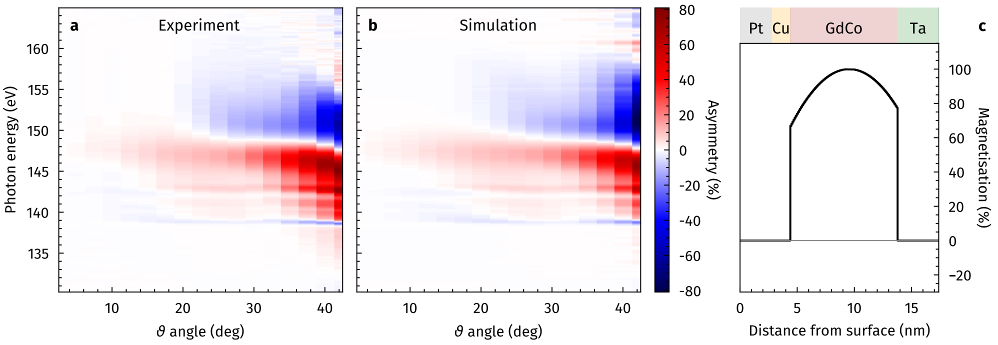

Supplementary Fig. 3 Static angle-resolved transverse magneto-optical Kerr effect (TMOKE) spectroscopy and simulations. a, Static angle-resolved TMOKE asymmetry (colour map) of the studied $Gd_{25}Co_{75}$ sample measured in the photon energy range across the Gd $N_{5,4}$ resonance. **b**, Simulation of the angle-resolved TMOKE asymmetry fitted to the experimental data. **c**, Equilibrium magnetisation depth profile determined from the fit.

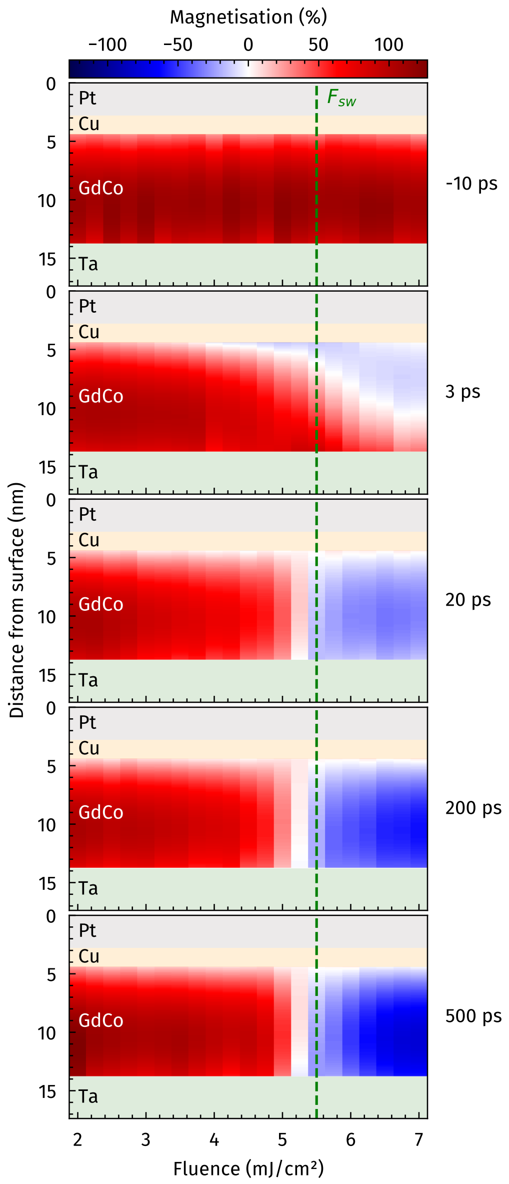

Supplementary Fig. 4 Fluence-dependent magnetisation depth profiling. Fluence-dependence of the transient magnetisation distribution (colour map) within the $Gd_{25}Co_{75}$ layer, comparing different times before and after excitation. The magnetisation depth profiles are obtained by fitting transient transverse magneto-optical Kerr effect (TMOKE) spectroscopy data recorded at fixed pump–probe delays (-10, 3, 20, 200 and 500 ps) as a function of excitation fluence (2.0–7.0 mJ/cm²). The data indicates the threshold fluence $F_{sw} = 5.5\,\mathrm{mJ/cm^2}$ (dashed green line), which is required for a full reversal of the $Gd_{25}Co_{75}$ layer magnetisation.

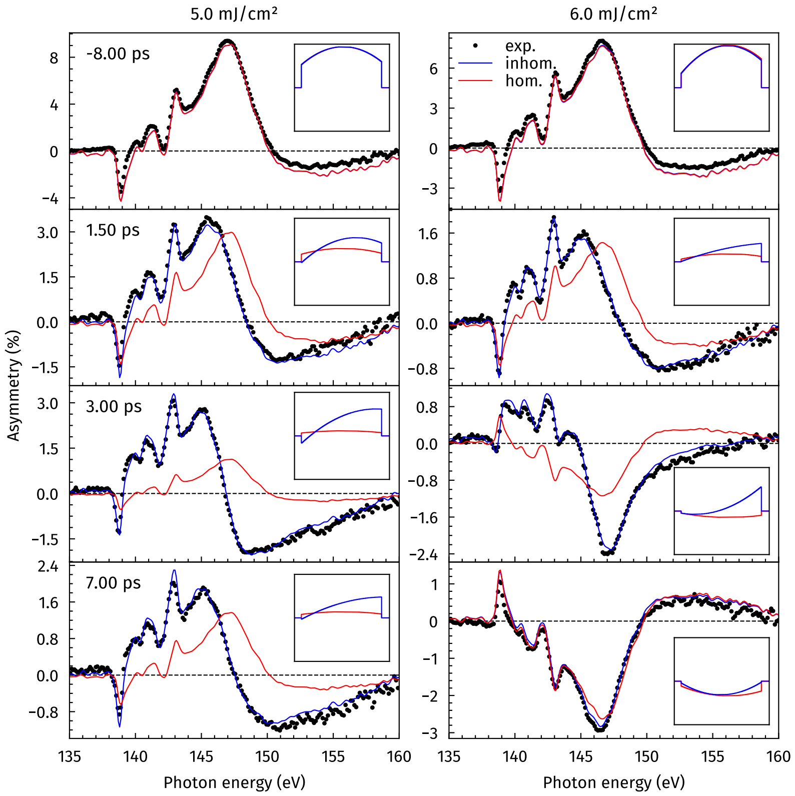

Supplementary Fig. 5 Inhomogeneous vs. homogeneous simulations. Simulations of the magnetic transverse magneto-optical Kerr effect (TMOKE) asymmetry fitted to the experimental data recorded for 5.0 and 6.0 mJ/cm^2 excitation by either allowing an inhomogeneous change of the magnetisation depth profiles (blue curves) or by constraining the fit to homogeneous dynamics, fitting only a factor that scales the equilibrium magnetisation profile obtained at negative delays (red curves). The insets show the corresponding magnetisation depth profiles.

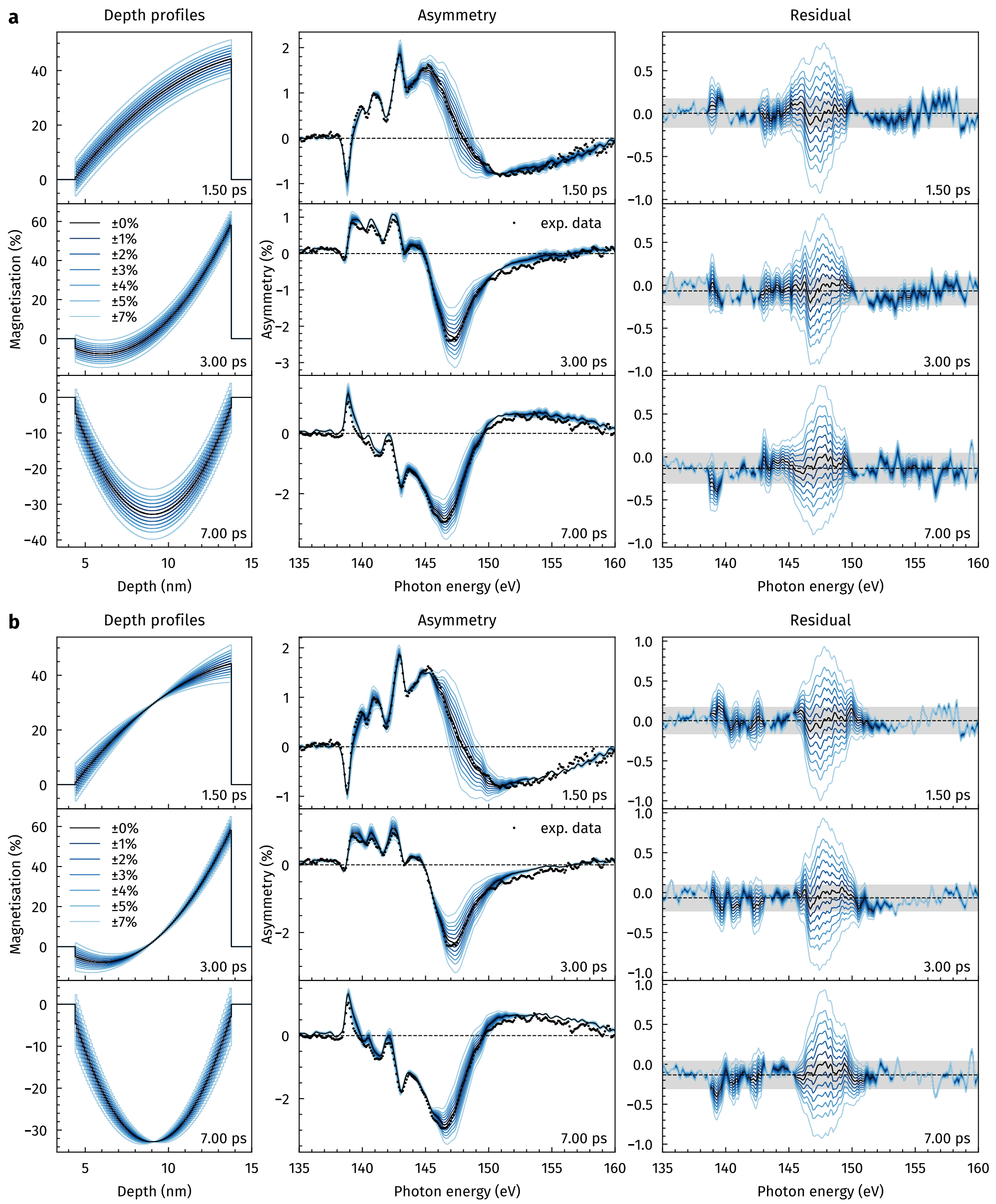

Supplementary Fig. 6 Significance analysis. Simulating the impact of adding artificial **a**, offsets and **b**, slopes (±1–7 %) to the best fits (denoted as ±0 %) of the magnetisation depth profiles on the transverse magneto-optical Kerr effect (TMOKE) asymmetry, analysing the corresponding residuals between simulated asymmetry and experimental data. The shaded areas correspond to a 95 % confidence interval (2σ of the best fit residual). The analysis is performed for three specific times (upper, mid and lower panels) after 6.0 mJ/cm^2 excitation: when the boundary between switched and not switched regions starts to emerge at the top, when it has propagated halfway through the $Gd_{25}Co_{75}$ layer and when it has reached the bottom. Note that for deviations larger than 2 % from the fitted magnetisation profiles, the residual gets larger than the confidence interval.

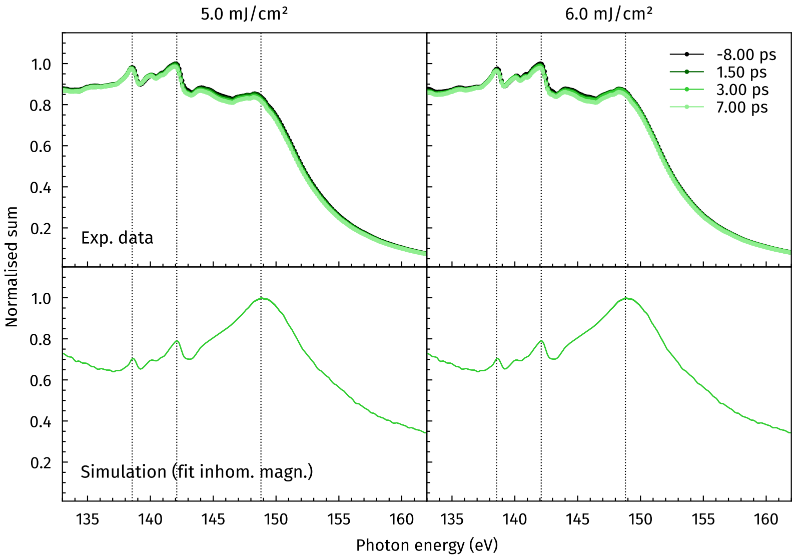

Supplementary Fig. 7 Transient sum reflectivity. Time-resolved (non-magnetic) reflectivity spectra normalised to the maximum before excitation (negative delay) as a function of excitation fluence and time after excitation (different shades of green). The experimental data (upper panels) and the simulations assuming inhomogeneous magnetisation dynamics (lower panels) are, within the experimental uncertainty, nearly constant over delay time. Please note that the experimental reflectivity spectra are influenced by the envelope of the incident spectrum of the radiation source. While this does not allow a fully quantitative comparison to the simulated spectra, it is not influencing the transverse magneto-optical Kerr effect (TMOKE) asymmetry, which is normalised to the sum reflectivities.

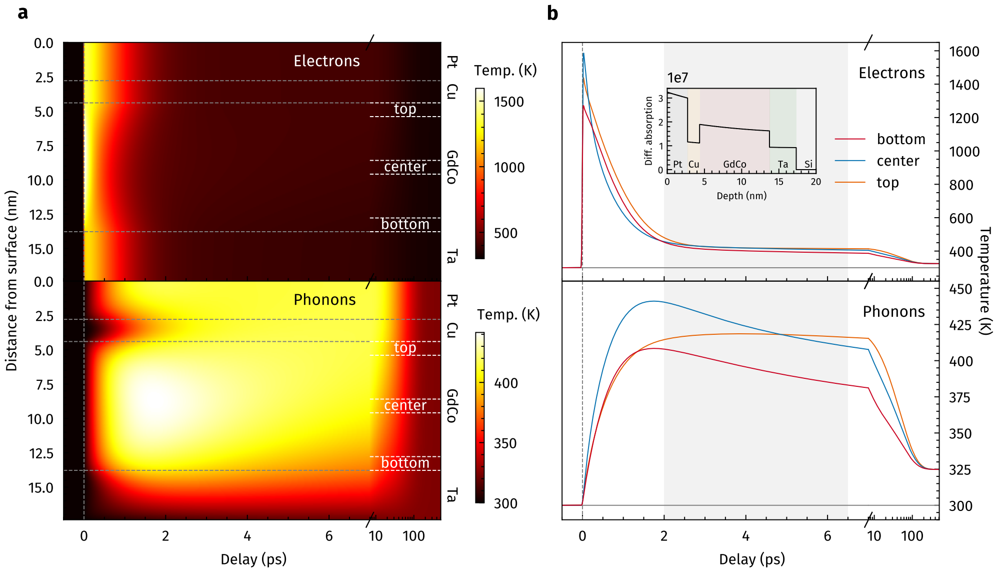

Supplementary Fig. 8 Transient evolution of electron and phonon temperatures. **a**, Electron and phonon temperature distribution (colour map) within the sample as a function of pump–probe delay, obtained from a diffusive two-temperature model (2TM) simulation with 6.0 mJ/cm^2 excitation fluence. **b**, Spatially integrated electron and phonon temperature, comparing the heat dynamics within 1 nm thin regions at the top, centre and bottom of the $Gd_{25}Co_{75}$ layer as indicated in panel **a**. The inset shows the depth-dependent differential absorption of the 2.1 µm pump pulse, which is incident from the left side on the Pt layer. For comparison, the grey shaded area taken from Fig. 4b shows the time interval during which the $Gd_{25}Co_{75}$ layer is transiently split into oppositely magnetised regions.

Layer	$\gamma\left(\frac{\mathrm{mJ}}{\mathrm{kg\,K^2}}\right)$	$c_\mathrm{p}\left(\frac{\mathrm{J}}{\mathrm{kg\,K}}\right)$	$k_\mathrm{e}^0\left(\frac{\mathrm{W}}{\mathrm{m\,K}}\right)$	$k_\mathrm{p}\left(\frac{\mathrm{W}}{\mathrm{m\,K}}\right)$	$G_\mathrm{ep}\left(\frac{\mathrm{W}}{\mathrm{m^3\,K}}\right)$
Pt [3]	34.6	133	66	5	$4 \cdot 10^{17}$
Cu [3]	11.2	384	396	5	$6.3 \cdot 10^{16}$
GdCo [4]	28.5	279	2.91	6.6	$5 \cdot 10^{17}$
Ta [3]	22.8	140	52	5	$1 \cdot 10^{17}$
Si	–	712 [5]	–	148 [6]	–

Layer	n	κ
Pt [7]	5.0	6.8
Cu [8]	0.94	14.1
GdCo [9]	2.7	8.3
Ta [10]	1.1	11.8
Si [11]	3.45	0

Supplementary Table 1 Parameters used in the diffusive two-temperature model (2TM) calculations. γ and c_p correspond to the material-dependent electronic heat capacity coefficient and phonon specific heat capacity, k_e^0 and k_p to the respective (equilibrium) thermal conductivities, and G_ep to the electron-phonon coupling constant. The γ and c_p values are provided in units of specific heat capacity, i.e., in relation to the mass of the material. n and κ correspond to the real and imaginary parts of the layer-dependent complex refractive index at 2.1 µm wavelength, used to calculate the depth-dependent laser absorption profile. The values of n and κ of the $Gd_{25}Co_{75}$ alloy are approximated by the refractive index of pure Co. Unless stated otherwise, all parameters within a row are taken from the reference specified in the first column.

References

[1] Zaumseil, P. *RCRefSimW – version 1.09* (developed at Leibniz Institute for High Performance Microelectronics (IHP), Frankfurt (Oder), Germany, 2009). URL https://www.ihp-microelectronics.com/fileadmin/pdf/services/Overview_of_Analytical_Methods.pdf.

[2] Henke, B., Gullikson, E. & Davis, J. X-ray interactions: Photoabsorption, scattering, transmission, and reflection at E = 50-30,000 eV, Z = 1-92. *Atomic Data and Nuclear Data Tables* **54**, 181–342 (1993). URL https://www.sciencedirect.com/science/article/pii/S0092640X83710132.

[3] Pudell, J.-E. *et al.* Heat transport without heating?—an ultrafast x-ray perspective into a metal heterostructure. *Advanced Functional Materials* **30**, 2004555 (2020). URL https://doi.org/10.1002/adfm.202004555.

[4] Steinbach, F. *et al.* Exploring the fundamental spatial limits of magnetic all-optical switching. *Nano Letters* **24**, 6865–6871 (2024). URL https://doi.org/10.1021/acs.nanolett.4c00129.

[5] Lide, D. R. (ed.) *CRC Handbook of Chemistry and Physics, 88th Edition* (CRC Press, Boca Raton, Florida, 2007).

[6] Ho, C. Y., Powell, R. W. & Liley, P. E. Thermal conductivity of the elements. *Journal of Physical and Chemical Reference Data* **1**, 279–421 (1972). URL https://doi.org/10.1063/1.3253100.

[7] Rakić, A. D., Djurišić, A. B., Elazar, J. M. & Majewski, M. L. Optical properties of metallic films for vertical-cavity optoelectronic devices. *Applied Optics* **37**, 5271–5283 (1998). URL https://doi.org/10.1364/AO.37.005271.

[8] Ordal, M. A., Bell, R. J., Alexander, R. W., Long, L. L. & Querry, M. R. Optical properties of fourteen metals in the infrared and far infrared: Al, Co, Cu, Au, Fe, Pb, Mo, Ni, Pd, Pt, Ag, Ti, V, and W. *Applied Optics* **24**, 4493–4499 (1985). URL https://doi.org/10.1364/AO.24.004493.

[9] Werner, W. S. M., Glantschnig, K. & Ambrosch-Draxl, C. Optical constants and inelastic electron-scattering data for 17 elemental metals. *Journal of Physical and Chemical Reference Data* **38**, 1013–1092 (2009). URL https://doi.org/10.1063/1.3243762.

[10] Ordal, M. A., Bell, R. J., Alexander, R. W., Newquist, L. A. & Querry, M. R. Optical properties of Al, Fe, Ti, Ta, W, and Mo at submillimeter wavelengths. *Applied Optics* **27**, 1203–1209 (1988). URL https://doi.org/10.1364/AO.27.001203.

[11] Li, H. H. Refractive index of silicon and germanium and its wavelength and temperature derivatives. *Journal of Physical and Chemical Reference Data* **9**, 561–658 (1980). URL https://doi.org/10.1063/1.555624.
