# Supplementary material for: Transient domain boundary drives ultrafast magnetisation reversal
Source: Nat Commun. 2025 Sep 5;16:8233. doi: 10.1038/s41467-025-63571-3 (PMC12413464; doi:10.1038/s41467-025-63571-3)
Supplement: Supplementary file 2 — Description of Additional Supplementary Files [file 41467_2025_63571_MOESM2_ESM.pdf]

Description of Additional Supplementary Files

Supplementary Movie 1. Animation of the depth-resolved magnetisation switching dynamics (colour map), comparing excitation below (5.0 mJ/cm²) and above (6.0 mJ/cm²) the threshold fluence for all-optical magnetisation switching of the entire $Gd_{25}Co_{75}$ layer.
